# Design of Digital Twin Cutting Experiment System for Shearer

**DOI:** 10.3390/s24103194

**Published:** 2024-05-17

**Authors:** Bing Miao, Yunwang Li, Yinan Guo

**Affiliations:** 1 School of Mechanical and Electrical Engineering, China University of Mining and Technology (Beijing), Beijing 100083, China; 2 Key Laboratory of Intelligent Mining Robotics, Ministry of Emergency Management, Beijing 100083, China; 3 China Academy of Safety Science and Technology, Beijing 100012, China

**Keywords:** shearer, similarity theory, digital twin, cutting experiment system

## Abstract

This study presents an advanced simulated shearer machine cutting experiment system enhanced with digital twin technology. Central to this system is a simulated shearer drum, designed based on similarity theory to accurately mirror the operational dynamics of actual mining cutters. The setup incorporates a modified machining center equipped with sophisticated sensors that monitor various parameters such as cutting states, forces, torque, vibration, temperature, and sound. These sensors are crucial for precisely simulating the shearer cutting actions. The integration of digital twin technology is pivotal, featuring a real-time data management layer, a dynamic simulation mechanism model layer, and an application service layer that facilitates virtual experiments and algorithm refinement. This multifaceted approach allows for in-depth analysis of simulated coal cutting, utilizing sensor data to comprehensively evaluate the shearer’s performance. The study also includes tests on simulated coal samples. The system effectively conducts experiments and captures cutting condition signals via the sensors. Through time domain analysis of these signals, gathered while cutting materials of varying strengths, it is determined that the cutting force signal characteristics are particularly distinct. By isolating the cutting force signal as a key feature, the system can effectively distinguish between different cutting modes. This capability provides a robust experimental basis for coal rock identification research, offering significant insights into the nuances of shearer operation.

## 1. Introduction

While new clean energy technologies are experiencing rapid development, fossil energy continues to serve as the predominant global energy source. In 2022, there was a 1% increase in global energy demand compared to the previous year, with fossil fuels contributing 82% of the total energy supply. China’s overall energy consumption rose to 5.41 billion metric tons of standard coal in 2022, marking a 2.9% increase from the preceding year. Longwall mining utilizing a shearer stands out as the most widely utilized method among underground coal mining techniques. The efficiency and productivity of longwall mining operations hinge upon the cutting performance of the shearer along the longwall face [1,2].

As a crucial component of the fully mechanized mining system, the shearer plays a key role in enabling efficient and concentrated coal extraction processes. This advancement has notably diminished the frequency of safety incidents in coal mines and enhanced the occupational environment for miners. The successful deployment of intelligent control mechanisms for the shearer is essential for the potential automation of fully mechanized mining operations. Consequently, the technology for distinguishing between coal and rock has been the subject of widespread study and focus [3,4,5]. Current methods for identifying coal and rock primarily utilize the operational signals produced during the shearer’s cutting of various coal and rock formations for classification purposes [6,7]. Thus, to yield significant insights, the experimental systems designed to simulate the shearer’s cutting process must adhere to specific standards of resemblance and dependability.

The methodologies employed by researchers to conduct experimental investigations for coal and rock identification predominantly encompass:

- In situ experiments, where sensors and measurement instruments are deployed directly on the mining face to gather operational signals from coal and rock for subsequent analysis and evaluation [8];
- Surface experiments, involving the use of an actual coal mining apparatus to perform real-life cutting tests on coal and rock on the surface, thereby collecting a range of performance metrics [9,10,11];
- Simulating shearer drum cutting tests, wherein scholars have devised and fabricated a simulating shearer drum apparatus to replicate the cutting actions on coal and rock materials [12,13,14];

In situ collection directly at the coal mining face guarantees the authenticity of the data acquired, yet it is subject to the constraints of a high-risk and severe work setting. Concurrently, devices used for signal collection must comply with explosion-proof standards, which escalates the experimental risk. Conducting experiments on the ground necessitates the availability of coal mining machinery, genuine coal and rock mediums, and pertinent apparatus, leading to relatively high testing expenses. On the other hand, the simulating shearer drum cutting experiment offers a cost-efficient approach to simulation experiments, although the reliability of this system remains a topic that warrants further examination. The simulating shearer drum experiment serves as an effective tool for analyzing and investigating the diverse information produced during the coal mining process, particularly in the study of coal and rock identification. For the precise replication of the shearer’s operational state while cutting, both the experimental apparatus and the simulation materials, constructed based on similarity theory, must closely resemble the original model. Currently, comprehensive and systematic research on experiments for identifying coal and rock is lacking.

**Figure 1:**
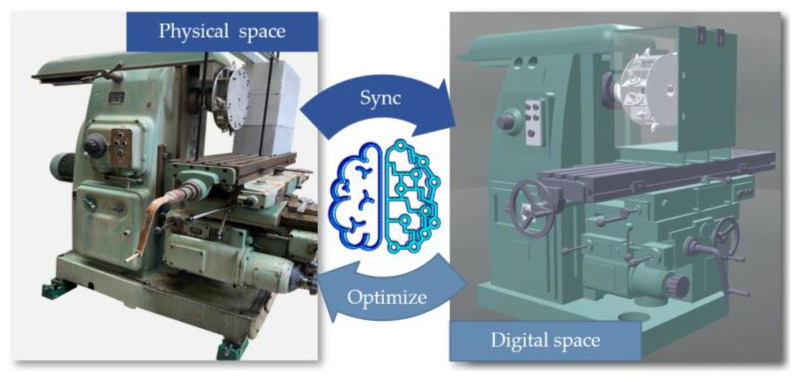
The physical space and the digital space.

Digital twin technology involves creating a virtual model of a physical entity in a digital format, enabling a bidirectional mapping, dynamic interaction, and real-time connection between the physical and digital spaces, as shown in Figure 1. Through digital twins, the attributes, structure, state, performance, functionalities, and behaviors of physical entities are mapped into the digital world, forming highly realistic dynamic multi-dimensional, multi-scale, and multi-physical models. The evolution of the digital twin is divided into three stages [15]: the virtual model stage, the basic digital twin stage, and the adaptive digital twin stage. In the first stage, we achieved digital twin modeling of the physical entity; in the second stage, we achieved data intercommunication between the twin and the physical domain, including data transfer and interaction between different digital twin models as well as between the digital model and the physical model; the third stage is the adaptive digital twin, aimed at achieving precise prediction of the physical entity by the digital twin and full-process collaborative optimization control. Therefore, drawing on existing research and simulation of similar theoretical principles, this paper outlines the development and construction of an experimental system for digital twin cutting of coal mining machines, aiming to collect performance data and realize the first two stages of digital twinning.

**Figure 2:**
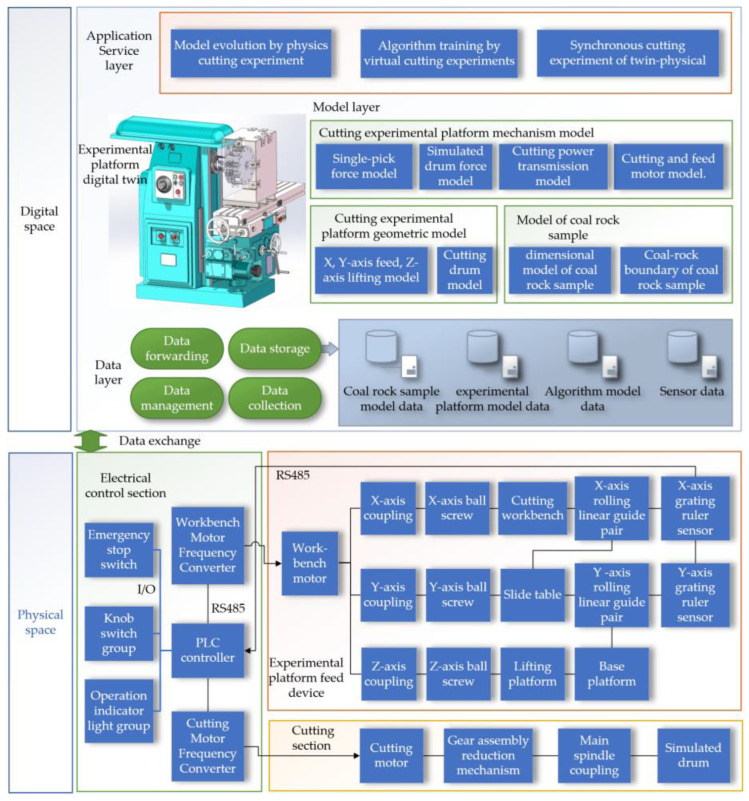
Digital Twin Cutting Experiment System Block Diagram.

The digital twin cutting system is primarily divided into physical and digital spaces, with data exchange between the two spaces facilitated through IoT technology. The physical space consists of three parts: the cutting section, the experimental platform feed device, and the electrical control section. The experimental platform is modified from the machining center, with major modifications including the design of a simulated drum; a cutting state detection sensor system; and the addition of three-dimensional cutting force, drum torque, vibration, and sound sensors. A grating scale sensor has been added to the feed device section of the experimental platform. The electrical control section has been enhanced with a worktable motor inverter and a cutting motor inverter, enabling speed control of these motors. The digital twin cutting experiment system block diagram is shown in Figure 2.

**Figure 3:**
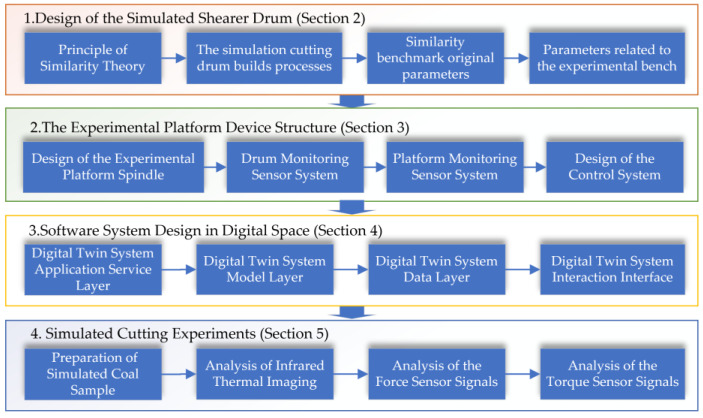
Digital Twin Cutting Experiment System Design Process Diagram.

The digital space comprises the data layer, the model layer, and the application layer, including the visualization of the experimental platform’s digital twin. The data layer is responsible for data forwarding, storage, management, and collection, covering coal rock sample data, experimental platform operational data, algorithm model data, and sensor data. The mechanism model layer includes the mechanism model, the geometric model of the cutting experimental platform, and the coal rock sample model. The application layer involves model evolution physical cutting experiments, algorithm training virtual cutting experiments, and physical synchronous cutting experiments with the digital twin. The design process diagram of the digital twin cutting experiment system is shown in Figure 3.

## 2. Design of the Simulated Shearer Drum

The design of the simulated drum is the key to the entire experimental system. Only by following the similarity theory can we ensure the similarity between the cutting of the simulated drum and the cutting of the coal mining machine in the coal mine.

### 2.1. Principle of Similarity Theory

The similarity π theory can be stated as: “For a physical system that contains n physical quantities and k fundamental dimensions, these n physical quantities can be expressed as a functional relationship among (n − k) independent similarity criteria π_1_, π_2_, …, π_n−k_”. This means that any physical equation:

(1) $$f\left(x_{1},x_{2},x_{3},\ldots ,x_{n}\right)=0$$

can be rewritten according to the similarity π theory as:

(2) $$\psi \left(\pi_{1},\pi_{2},\pi_{3},\ldots ,\pi_{n-k}\right)=0$$

Through this transformation, the original physical equation is converted into a criterion relation, simplifying the problem. When the prototype and the model are similar, if the similarity criteria maintain the same value at corresponding points and corresponding moments, then their π relations should also be identical, that is:

(3) $$\left\{\begin{array}{l} \psi {\left(\pi_{1},\pi_{2},\pi_{3},\ldots ,\pi_{n-k}\right)}_{p}=0\\ \psi {\left(\pi_{1},\pi_{2},\pi_{3},\ldots ,\pi_{n-k}\right)}_{m}=0 \end{array}\right.$$

The second similarity theorem indicates that in mutually similar phenomena, the similarity criteria do not need to be derived using similarity indicators. As long as the relationship equations of various physical quantities are converted into the form of dimensionless equations, the terms of these equations are the similarity criteria.

p_i_ is used to represent the ith physical quantity in a system, and m_i_ to represent the corresponding physical quantity in another system (a similar system). The ratio of these two physical quantities is called the similarity coefficient (or transformation coefficient), denoted as C_i_:

(4) $$C_{i}=\frac{p_{i}}{m_{i}}$$

Equation (4) indicates that every physical quantity of a system is converted into the corresponding physical quantity in another system through the linear transformation of the parameter C_i_. In the transformation, the transformation coefficients C_i_ for different physical quantities (such as the modulus of elasticity E and length L) can be different, but within the set of similar systems, each transformation coefficient C_i_ is strictly constant. In similarity analysis, different similarity coefficients C_i_ play the role of assigning values to different physical quantities (including geometric quantities). The choice of similarity coefficients C_i_ depends on the nature of the problem under study and experimental conditions, among other factors. Moreover, the similarity coefficients are constant in two similar systems but have different values for a third system that is similar to these two systems.

### 2.2. Design of the Simulated Drum

**Figure 4:**
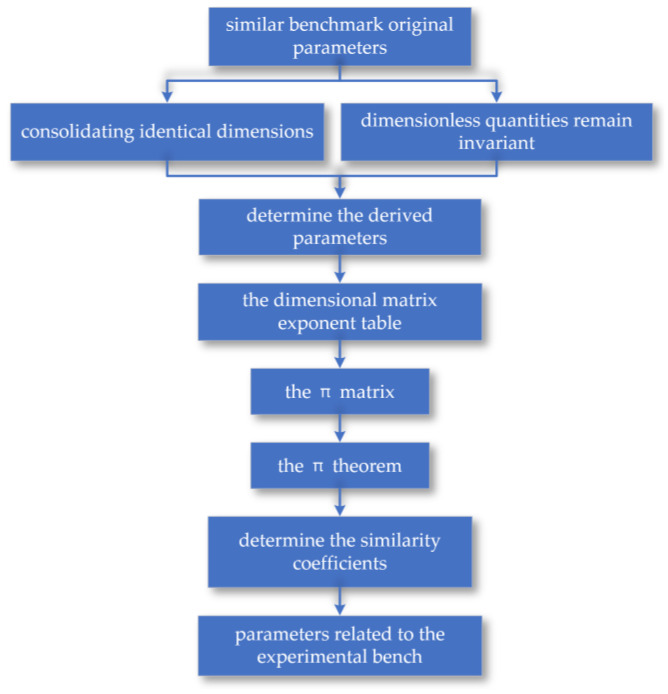
The simulation cutting drum builds processes based on similarity theory.

As the core cutting component in the coal mining machine’s cutting process, designing a similar simulation around the cutting drum is key to ensuring the experimental system and the prototype machine’s working conditions are similar. The simulation cutting drum builds processes based on similarity theory as shown in Figure 4. Therefore, this thesis focuses on the drum structure of the coal mining machine as the main research subject, derives similarity criteria through MLT dimensional analysis, and studies the cutting mechanism of the drum during operation, as well as the related motion and structural parameters.

Parameters defining the drum’s geometric structure as Table 1 include its overall diameter (D), the external diameter of the blade (D_y_), the depth of cut made by the drum (B), the angle of elevation for the spiral blade (α_y_), the leading distance of the blade (L), the total number of blade heads (Z), the spacing between blades (S_y_), the angle of wrap for the blade around the hub (β_y_), the spacing of the picks (T_c_), the angle at which the picks are mounted (γ), and the angle of pick inclination (λ_s_). For material parameters, the focus lies on simulating the compressive strength (σ) and the density (ρ) of coal and rock, in line with the criteria for coal and rock identification. Operational parameters encompass the range of the swing angle (θ) for the rocker arm, the rotational velocity of the drum (n), and the speed of traction (v) [16].

Using the MLT basic dimensional system (i.e., mass, length, time), the values and dimensions of the relevant parameters of the prototype are listed. In the design of similar models, dimensionless physical quantities have the same values between the prototype and the model, making it unnecessary to derive related similarity criteria. From the analysis above, it is necessary to derive similarity criteria for a total of five parameters as Table 2: D, n, ρ, v, σ. According to the second theorem of similarity (the π theorem), with five similarity parameters and three basic dimensions, the number of π rules is two, calculated as 5-3. Dimensional analysis shows that D, n, ρ include the three basic dimensions of M, L, T, and the determinant formed by them is not zero. They correspond, respectively, to parameters related to the structure of the cutting part, the motion parameters of the coal mining machine, and the characteristics of the cutting material. Therefore, these are selected as the basic physical quantities to list in the dimensional matrix exponent table.

Use the exponential method to analyze the dimensions of the system and obtain the linear homogeneous equations of the quality system.

(5) $$\left\{\begin{array}{l} {M:a}_{2}+a_{5}=0\\ L:-3a_{2}+a_{3}+a_{4}-a_{5}=0\\ T:-a_{1}-a_{4}-2a_{5}=0 \end{array}\right.$$

The similarity criterion for calculating different dimension parameters is:

(6) $$\left\{\begin{array}{l} \pi_{1}=\frac{v}{\mathrm{nD}}\\ \pi_{2}=\frac{\sigma }{n^{2\;}\rho D^{2}} \end{array}\right.$$

The π term is an invariant, and the similarity index is one according to the first similarity theorem. The similarity coefficient expression is as follows:

(7) $$\left\{\begin{array}{l} C_{n}C_{D}=C_{v}\\ C_{\rho }C_{v}^{2}=C_{\sigma } \end{array}\right.$$

The similarity coefficient for drum diameter, C_D_, requires that the model drum diameter be ≤400 mm, therefore C_D_ is set to 1/3 as Table 3. The simulated cutting material is made from coal dust and rock dust cut by the prototype machine, hence C_ρ_ equals 1.

**Figure 5:**
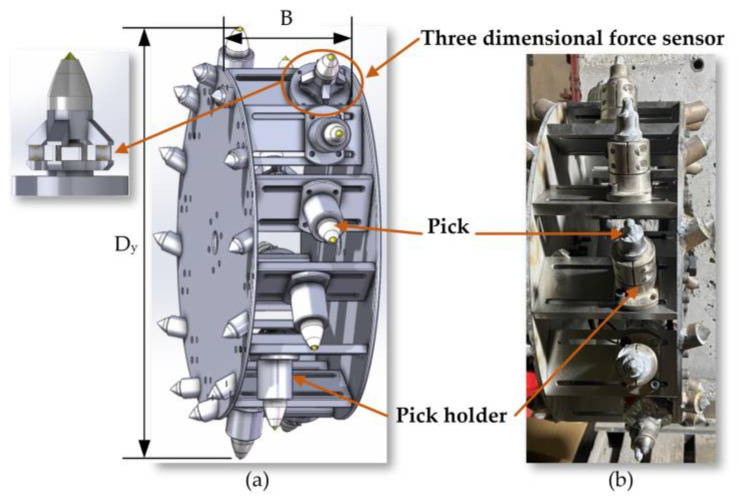
(**a**) The model of the cutting drum; (**b**) the physical cutting drum.

Based on the structure of the prototype shearer, it is determined that the simulated cutting drum is configured with teeth arranged in a sequential manner. The number of blade heads on the drum is set to two, corresponding to three cutting lines, with two teeth configured on each cutting line. Based on this, establish the model of the cutting drum, as shown in Figure 5.

## 3. The Device Structure of the Experimental Platform 

The physical experimental platform was constructed through a comprehensive modification of the machining center model machine tool, involving multiple key components: the base, bed, lifting platform, saddle, workbench, crossbeam, and tool post support, among others. The base provides fundamental support for the machine tool, with its stability being crucial to the overall work efficiency and precision of the machine; the bed serves as the main frame of the machine tool, supporting the installation of various parts. Positioned at the top of the bed, and connected through dovetail guides, the crossbeam is equipped with a drum support at its front, facilitating the installation of other tools or devices. The lifting platform enhances the flexibility and functionality of the machine tool by enabling vertical movement of the workbench through a vertical screw connected to the nut on the base. The workbench, including the rotary table and saddle, plays a key role in performing specific tasks and can accommodate a variety of work demands.

This modification focuses on three main aspects:

- The design and improvement of the spindle part—the primary task is to develop a spindle part suitable for the experimental device, including designing an appropriate drum device and connecting it to the machine tool’s spindle. This step is vital to ensuring that the machine tool can perform the required experimental operations.
- The integration of sensors and data acquisition systems—to accurately monitor and evaluate various parameters during the experimental process, the experimental device integrates efficient sensors and data acquisition systems. This includes measuring physical parameters such as force, temperature, and vibration, and involves the real-time collection, processing, and analysis of data to ensure the accuracy and reliability of experimental results.
- The development and application of the numerical control system—an advanced numerical control system has been developed to precisely control the spindle cutting motor and the workbench motor. This system is not only easy to use but also provides high precision and rapid response control, meeting the complex experimental requirements and changing work conditions.

Through these modifications, the physical experimental platform has significantly improved in flexibility, precision, and efficiency.

### 3.1. Design of the Experimental Platform Spindle

The main feature of the physical experimental platform is its complex main motion transmission system. This system transmits the rotary power of the cutting motor through a series of precisely configured transmission shafts (Shafts I to IV) to the main spindle. The spindle then drives the simulated cutting drum mounted on it to perform cutting actions, simulating the operation of a real coal mining machine. The power transmission process starts from the cutting motor and ends with the simulated drum, with multiple mechanical components working together to complete the power transmission and speed change.

In the initial stage, the power of the cutting motor is transmitted to Shaft I through a flexible coupling, ensuring that Shaft I rotates at the same speed as the motor. Shaft I uses a pair of fixed gear ratios to transmit power to Shaft II. Shaft II is equipped with a triple sliding gear device, which can provide three different speeds to Shaft III as needed. Similarly, the triple sliding gears on Shaft IV mesh with the gears on Shaft III, allowing Shaft IV to achieve three speeds based on the speed of Shaft III. Thus, Shaft IV can achieve nine different speed changes. Moreover, the double sliding gears at the right end of Shaft IV mesh with the gears on the spindle, allowing the spindle to reach eighteen different speeds to meet various cutting conditions.

**Figure 6:**
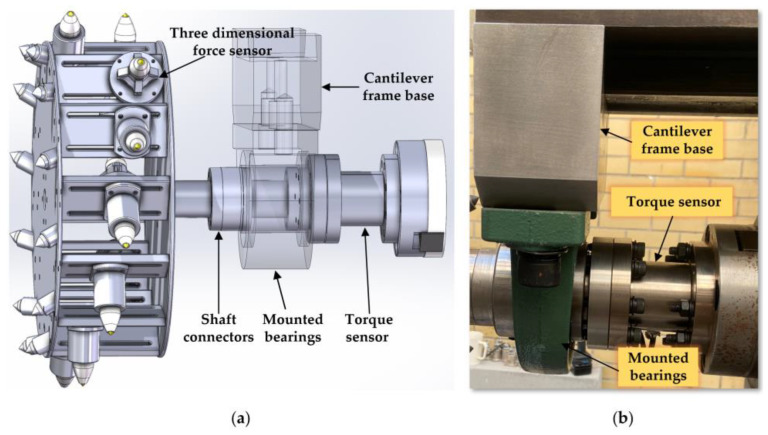
(**a**) The model of the platform spindle; (**b**) the physical platform spindle.

The spindle is a carefully designed hollow shaft, equipped with a special centering cone hole, end plane, and external cylindrical surface at the front end, along with two end-face keys, intended to ensure effective torque transmission and precise positioning of the equipment. The through-hole of the spindle is used for installing the tensioning tool rod and provides a pathway for the sensor cables on the drum to pass through to the rear signal collection device. To accurately monitor the cutting torque of the simulated drum, a torque sensor is installed between the spindle and the drum. To minimize the impact of tangential forces on the measurement, the sensor is externally equipped with a bearing seat, fixed to the top beam, effectively bearing tangential forces and ensuring accurate measurement. The flanges at both ends of the torque sensor are connected to the external cylindrical surface of the spindle and the simulated drum through a coupling, ensuring efficient power transmission and precise control. The model and physical structure of the platform spindle are shown in Figure 6.

### 3.2. Design of the Sensor and Acquisition System

**Figure 7:**
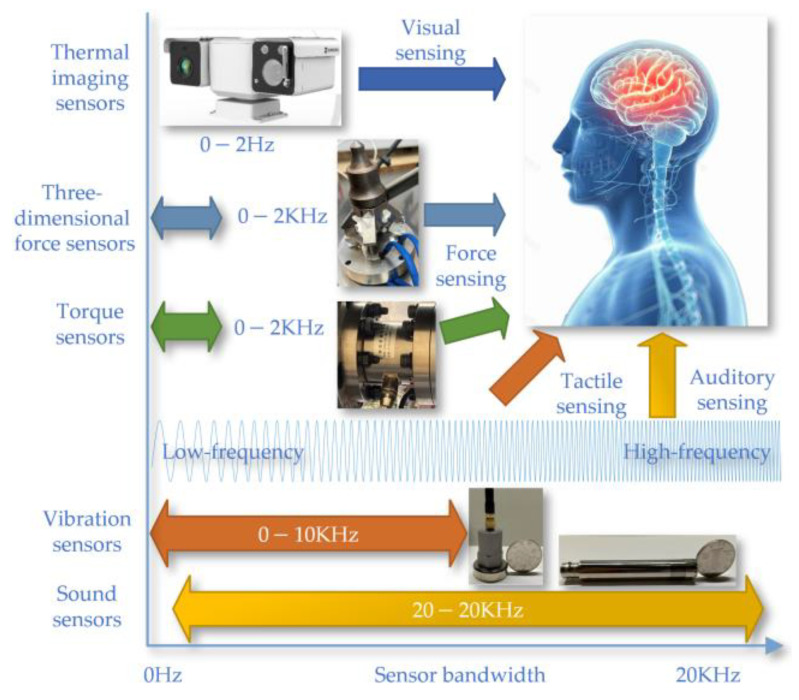
The sensors’ functioning and frequency responses.

This article illustrates the working principles and operational frequency bandwidths of various types of sensors on the experimental platform by analogizing the sensors’ functioning and frequency responses with the human body’s visual, tactile, and auditory models as shown in Figure 7. The experimental platform includes thermal imaging, three-dimensional force, torque, vibration, and sound sensors, which are used to detect visual, force, tactile, and auditory signals, respectively. The thermal imaging sensor operates within a frequency range of 0 to 2 Hz, mainly for visual signal detection. The three-dimensional force and torque sensors have a bandwidth of 0 to 2 kHz, used for force detection. The vibration sensor’s bandwidth ranges from 0 to 10 kHz, for tactile signal detection. The sound sensor operates over a wider bandwidth, from 20 to 20 kHz, for auditory signal detection. The differences in these bandwidths reflect the capabilities of the sensors to capture the respective physical signals.

The experimental platform utilizes multimodal sensors to comprehensively monitor the operation of the experimental platform, including temperature changes during the cutting process, three-dimensional force on the cutting teeth, drum torque, simulated drum vibration, cutting noise, drum rotation speed, worktable displacement, and cutting motor current. This monitoring network consists of a three-dimensional force sensor, a torque sensor, two grating scale sensors, an axial encoder sensor, three Hall current sensors, a vibration acceleration sensor, and a sound sensor, ensuring precise monitoring of the coal mining machine’s operational status. Through this system, researchers can accurately record and analyze the operational data of coal mining machines in a simulated environment, which is crucial for understanding the working principles of coal mining machines, identifying potential issues, optimizing design, and improving efficiency.

#### 3.2.1. Drum-Monitoring Sensor System

**Figure 8:**
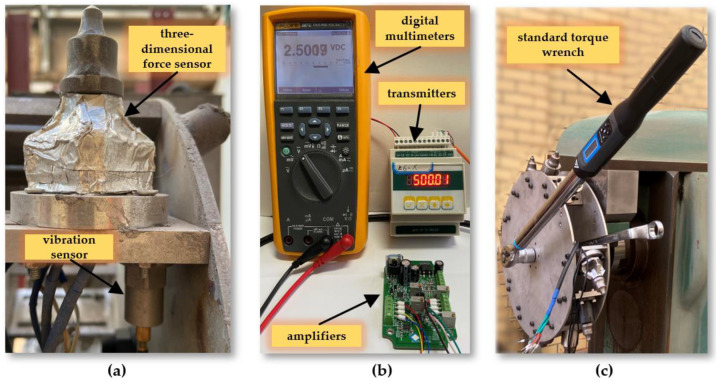
(**a**) Arrangement of sensors; (**b**) force sensor calibration; (**c**) torque sensor calibration.

The simulated drum parameter monitoring sensor system includes a cutter tooth three-dimensional force sensor, drum torque sensor, vibration sensor, and sensor acquisition system. The cutter tooth three-dimensional force sensor is specifically designed for coal and rock cutting conditions, capable of detecting the three-dimensional force on the cutter tooth, with X, Y, and Z signal output channels [17]. Each channel has its own independent signal collection circuit, ensuring no interference between any two channels and guaranteeing the sensor’s authenticity and reliability. In simulated cutting tests, it is necessary to measure the cutting torque of the multi-tooth experimental drum. The torque sensor is installed on the main drive shaft of the experimental stand to capture torque data during the cutting process, supporting the analysis of cutting performance. An IEPE vibration sensor is chosen for its strong anti-interference capability and wide frequency response range up to 15 KHz (±3 dB), with a measurement range of ±50 g. Sensors are connected to magnetic bases through threads, and magnetic bases are adhered to the surface with polishing glue. Both the cutter tooth three-dimensional force sensor and the drum torque sensor are based on the strain gauge principle and have undergone sensitivity and linearity checks before leaving the factory. To ensure data accuracy, the collection system still requires calibration, as shown in Figure 8.

**Figure 9:**
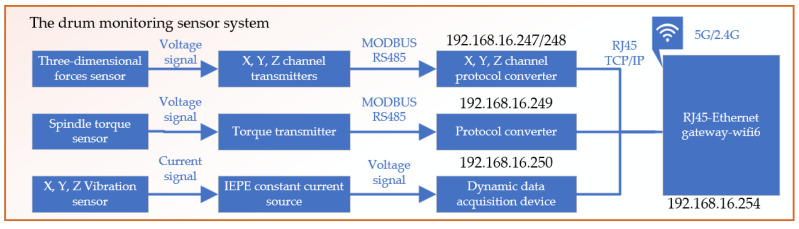
The Drum Signal Acquisition System Block Diagram.

To match the input signals of the selected data acquisition card, all of the sensors’ output signals were uniformly converted to voltage signals. Appropriate signal conditioners were chosen based on the characteristics of each signal for processing. Then, the sensors’ output voltage signals were transmitted to the computer through the data acquisition board. The hardware architecture of the experimental setup’s measurement is shown in Figure 9.

#### 3.2.2. Platform Monitoring Sensor System

**Figure 10:**
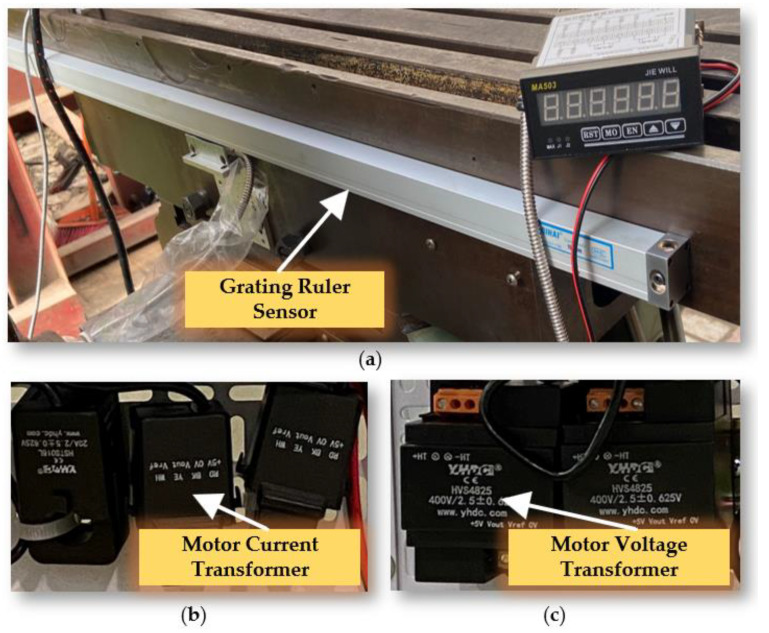
(**a**) Grating Ruler Sensor; (**b**) Motor Current Transformer; (**c**) Motor Voltage Transformer.

**Figure 11:**
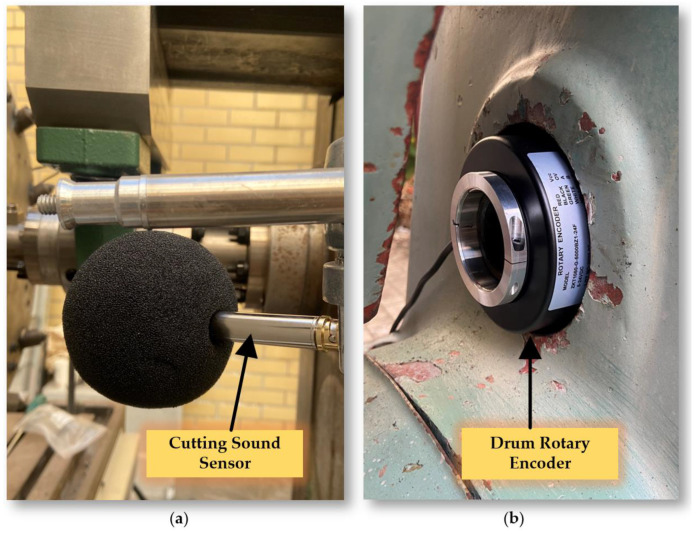
(**a**) Cutting Sound Sensor; (**b**) Drum Rotary Encoder.

The experimental platform sensor monitoring system is meticulously designed to capture and analyze a wide range of parameters that are essential for evaluating the performance and condition of mechanical systems. It includes several advanced components, as shown in Figure 10 and Figure 11.

Grating Ruler Sensor: Acquires high-precision positional information related to displacement, operating on precise measurements from a grating scale. This is crucial for tasks demanding high accuracy. Drum Rotary Encoder: Gathers data on the rotational angle of the main spindle, key for understanding the dynamics of drum rotation and providing insights into the spindle’s speed and direction. Cutting Sound Sensor: With an IEPE (integrated electronics piezoelectric) sound sensor, the system captures ambient noise, including sounds from cutting operations. Powered by a constant current source, it converts acoustic signals to voltage signals for noise analysis, useful in monitoring tool wear or detecting operational anomalies. Motor Current Transformer: Monitors electric currents across three channels (A, B, and C) of the motor, aiding in the detection of phase imbalances. Motor Voltage Transformer: Tracks the voltage signals from the inverter to the motor.

**Figure 12:**
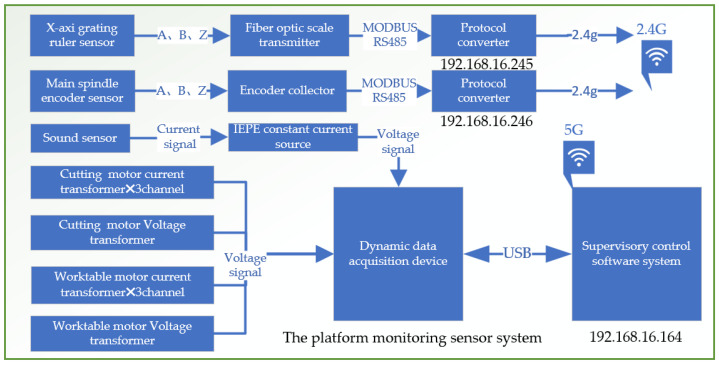
The Platform Signal Acquisition System Block Diagram.

Additionally, the system incorporates a data acquisition converter essential for converting analog signals into digital data for computer analysis, as shown in Figure 12. This feature enables comprehensive analysis of sensor data, supports real-time monitoring, and aids in post-operation evaluation.

### 3.3. Design of the Control System

Due to the design limitations of its spindle transmission system and feed transmission system, the machining center can only select from a few fixed gear ratios, which fails to meet the diverse requirements for spindle cutting speed and feed speed in cutting tests. Cutting tests are a crucial part of determining machining conditions, including choosing the optimal spindle speed and coal rock sample movement speed, to ensure high efficiency and precision in the machining process. To address this issue, a specialized numerical control (NC) system for the cutting experimental apparatus was developed. This system is designed to provide precise control over the cutting process, enabling adjustments to the spindle cutting speed and workpiece feed speed beyond the original fixed gear ratios of the machining center.

**Figure 13:**
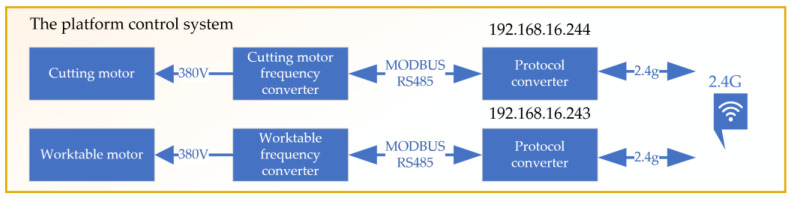
The Platform Control System Block Diagram.

**Figure 14:**
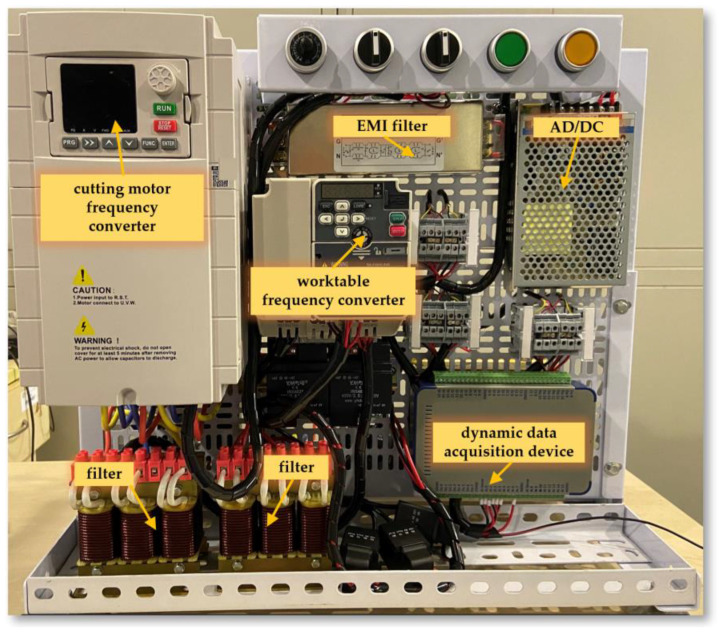
Electrical Control Device for the Cutting Experimental Platform.

The core of this NC system consists of two main parts: control of the spindle cutting motor and control of the worktable motor, as shown in Figure 13. The spindle cutting motor control is responsible for adjusting the cutting speed of the spindle, allowing for a wide and precise range of speeds. This flexibility is crucial for conducting cutting tests under various conditions to identify the most efficient cutting parameters. Similarly, the control of the worktable motor plays a key role in managing the feed speed of the workpiece. By precisely controlling the movement of the worktable, the system ensures that coal rock samples are fed at the optimal speed. The electrical control device for the cutting experimental platform is shown in Figure 14.

## 4. Software System Design in Digital Space

### 4.1. Application Service Layer

**Figure 15:**
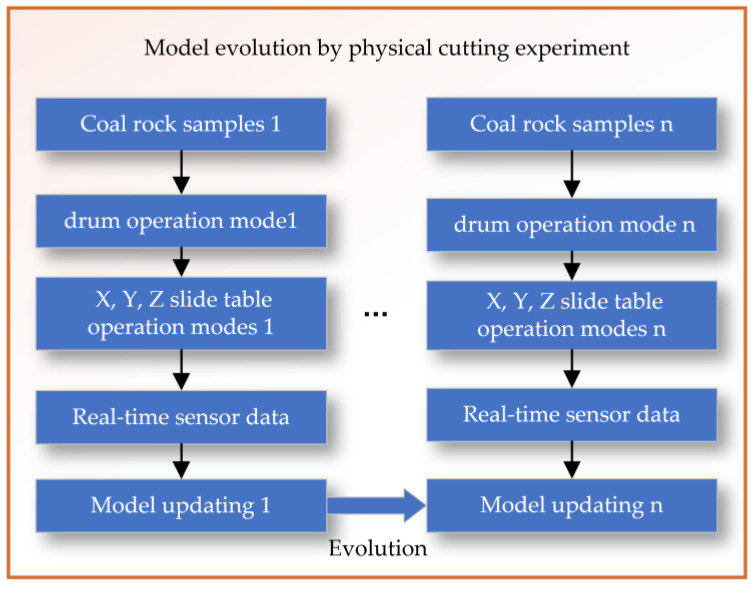
Model evolution by physical cutting experiment.

The evolution of the physical cutting experimental model is achieved by setting rollers with different rotation speeds and X, Y, Z sliding tables with different feed speeds to cut n groups of coal and rock samples with different hardness and distribution characteristics, as shown in Figure 15. This process allows for the collection of corresponding sensor data, which is then used to continuously revise and update the model of the cutting mechanism on the experimental platform.

**Figure 16:**
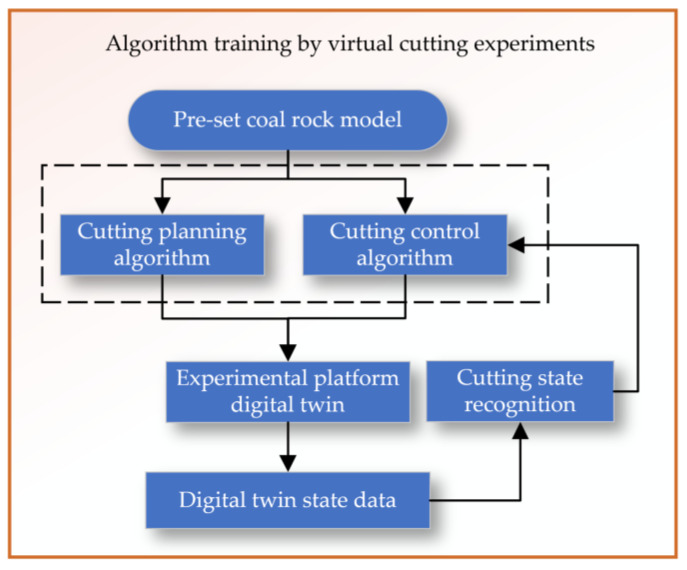
Algorithm training by virtual cutting experiments.

The planning and control algorithm training for virtual cutting tests begins with setting an initial coal and rock model, as shown in Figure 16. Then, based on the cutting planning and control algorithms, virtual cutting is conducted in a digital twin environment. By analyzing the state perception data of the digital twin, the cutting state is identified, and the cutting planning and control algorithms are adjusted accordingly. In the virtual environment, a closed-loop system of planning–control–cutting–feedback is formed, allowing for continuous optimization and updating of the planning and control algorithms.

**Figure 17:**
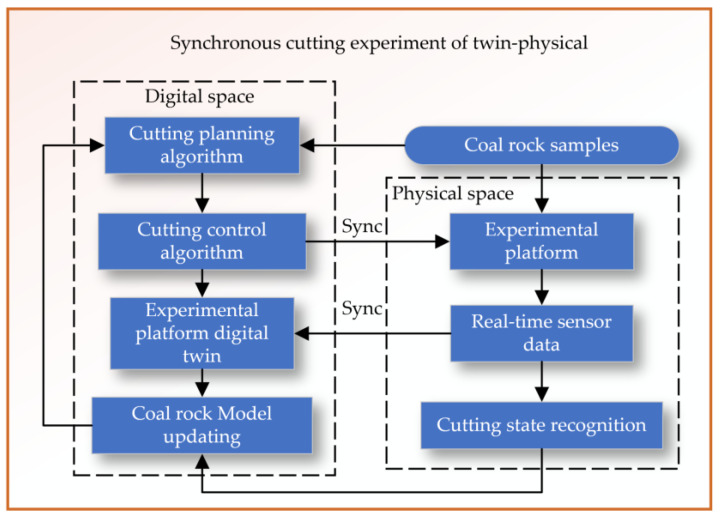
Synchronous cutting experiment of twin–physical system.

The synchronous cutting experiment of the twin–physical system is divided into two main areas: the digital space and the physical space, as shown in Figure 17. The digital space cutting planning algorithm refers to the initial stage where a digital algorithm is used to plan how the cutting of the coal rock samples will be executed. Cutting after planning, the control algorithm would be responsible for the actual execution of the cutting process in the digital twin system. The experimental platform digital twin represents a virtual replica of the physical experimental platform, where the cutting algorithms are tested. Based on feedback and results from the digital twin, the coal rock model is updated to reflect new insights or to improve the cutting process. The physical space is a set of actual physical samples that will be cut in the experiment. Experimental platform is the physical counterpart to the digital twin where the actual cutting takes place. Real-time sensors on the experimental platform provide real-time data on the cutting process. The system recognizes the state of the cutting process, using real-time sensor data.

The synchronization (labeled “Sync”) between the digital twin and the physical platform suggests that data and insights are shared between the two to ensure that the digital planning and control algorithms are accurate and reflective of the real-world physical cutting process. Overall, the system is designed to use a digital–physical twin approach to simulate, plan, and control the cutting of coal rock samples, aiming for optimization of the process and better prediction of outcomes.

### 4.2. Model Layer

The model layer mainly analyzes and stores the following models: the geometric model of the cutting experiment platform, the geometric model of the coal rock sample, and the mechanism model of the cutting experiment platform.

#### 4.2.1. Geometric Model

The geometric model refers to a mathematical representation method used to describe the shape and structure of objects. Geometric models are three-dimensional (such as solid objects) and aim to mathematically capture and express the geometric features of objects, enabling computers to process, analyze, render, and simulate them. Geometric models are usually represented by data structures consisting of vertices, edges, and faces, which can construct complex geometric models, from simple geometric bodies to highly detailed 3D models.

**Figure 18:**
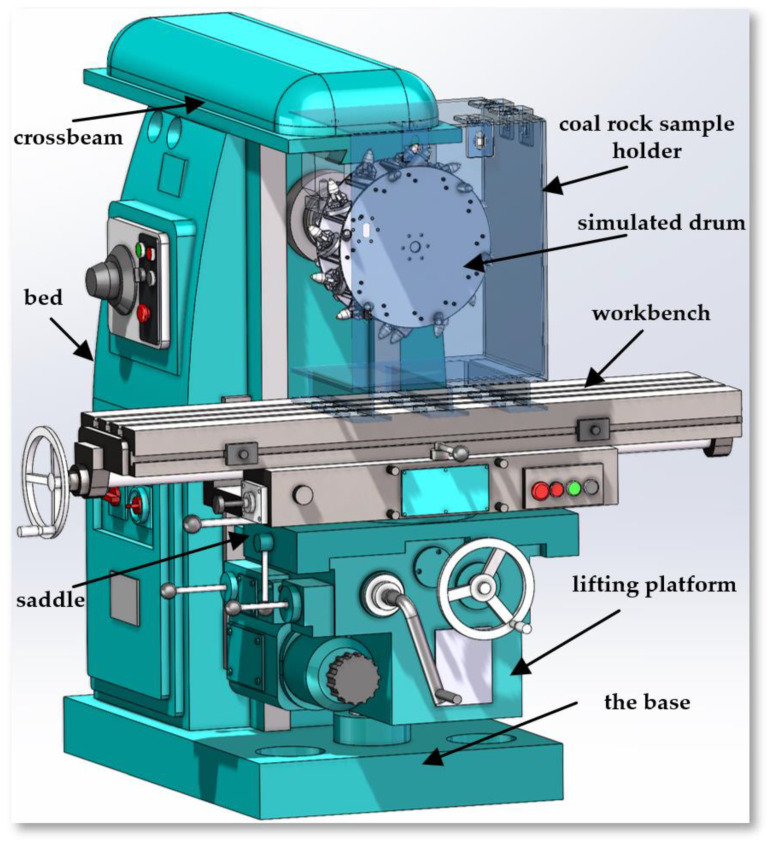
The geometric models of the cutting experiment platform.

The geometric models of the cutting experiment platform mainly include: X- and Y-axis feeding, the Z-axis lifting model, and the cutting unit model, as shown in Figure 18. X- and Y-axis feeding and the Z-axis lifting model describe the motion control of the cutting device in three orthogonal directions, including the adjustment of feeding speed and lifting speed. The cutting unit model refers to the design and functionality of a single cutting drum that completes the coal rock cutting task.

The coal rock sample model includes: the 3D model of the coal rock sample and the coal rock interface model of the coal rock sample. The 3D model of the coal rock sample represents the three-dimensional appearance of the coal rock sample, containing the shape, size, and internal structure of the coal rock. The coal rock interface model studies the properties of the interface between coal and rock, which is crucial for understanding mechanical behavior and cutting efficiency during the cutting process.

#### 4.2.2. Mechanism Model

The mechanism model is a type of model used to describe and explain the behavior of a phenomenon or system. It is based on an understanding of the system’s internal mechanisms, principles, and interactions, revealing the rules and processes of the system’s operation at a microscopic level. Mechanism models are usually established on the basis of a professional field, focusing on the components of the system and their interactions. They describe the dynamic characteristics and behavior of the system through mathematical equations, logical relationships, or graphics. Mechanism models emphasize an understanding of the internal mechanisms and processes of the system, offering stronger interpretability. The mechanism models of the cutting experiment platform include: the single-tooth force model, the simulated drum force model, the cutting power transmission model, and the cutting and feeding motor model.

The single-tooth force model focuses on the mechanical behavior and force conditions of a single cutting tooth during the coal rock cutting process. The simulated drum force model simulates the force generated by the drum during coal rock cutting, including the drum’s dynamic parameters and force. The cutting power transmission model analyzes the energy transfer path from the power source to the cutting head, and how the power is transmitted and acts on the coal rock.

**Figure 19:**
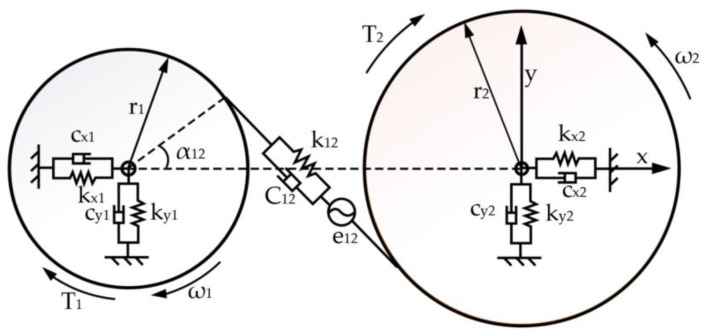
Translational–rotational model of a fixed-shaft gear set.

**Figure 20:**
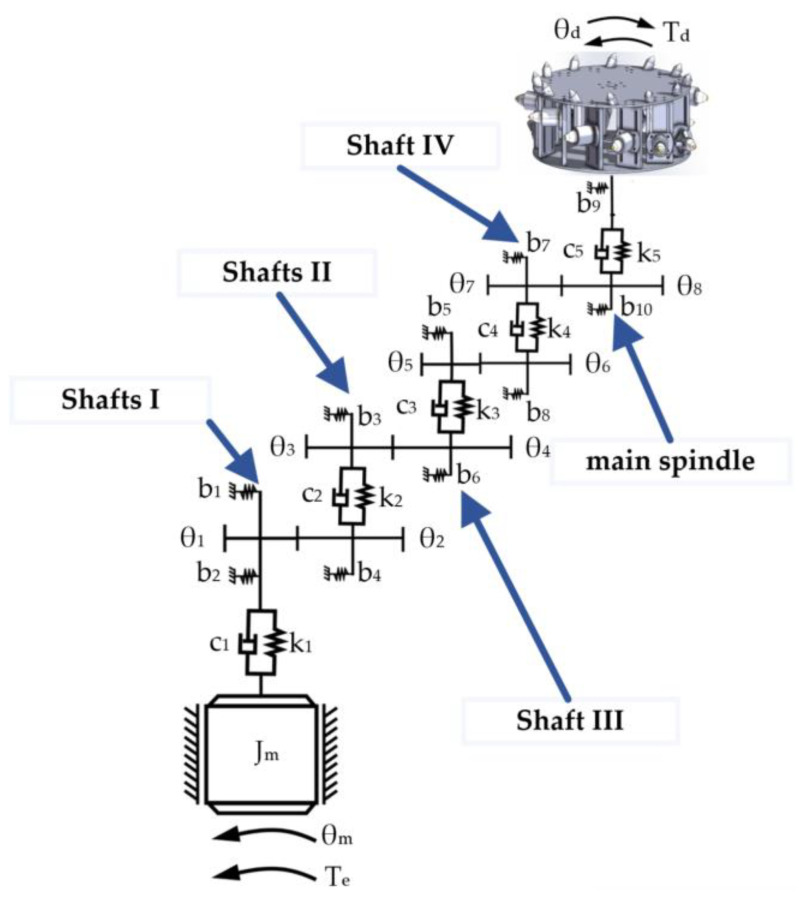
Lumped parameter model for the multistage gearbox.

This system transmits the rotary power of the cutting motor through a series of precisely configured transmission shafts (Shafts I to IV) to the main spindle. Taking a single-stage parallel-axis system as an example, the translational–rotational model is presented in Figure 19, and its corresponding dynamical equations are formulated as shown in Equation (8). The lumped parameter model for the multistage gearbox of the cutting experimental platform is shown in Figure 20.

(8) $$\left\{\begin{array}{l} m_{1}{\ddot{x}}_{1}=-\left(k_{12}\delta_{12}+c_{12}{\dot{\delta }}_{12}\right)\sin\alpha_{12}-k_{x1}x_{1}-c_{x1}{\dot{x}}_{1}\\ m_{1}{\ddot{y}}_{1}=\left(k_{12}\delta_{12}+c_{12}{\dot{\delta }}_{12}\right)\cos\alpha_{12}-k_{y1}y_{1}-c_{y1}{\dot{y}}_{1}\\ m_{2}{\ddot{x}}_{2}=\left(k_{12}\delta_{12}+c_{12}{\dot{\delta }}_{12}\right)\sin\alpha_{12}-k_{x2}x_{2}-c_{x2}{\dot{x}}_{2}\\ m_{2}{\ddot{y}}_{2}=-\left(k_{12}\delta_{12}+c_{12}{\dot{\delta }}_{12}\right)\cos\alpha_{12}-k_{y2}y_{2}-c_{y2}{\dot{y}}_{2}\\ J_{1}{\ddot{\theta }}_{1}=T_{1}-\left(k_{12}\delta_{12}+c_{12}{\dot{\delta }}_{12}\right)r_{1}\\ J_{2}{\ddot{\theta }}_{2}=\left(k_{12}\delta_{12}+c_{12}{\dot{\delta }}_{12}\right)r_{2}-T_{2}\\ \delta_{12}=\left(x_{1}-x_{2}\right)\sin\alpha_{12}-\left(y_{1}-y_{2}\right)\cos\alpha_{12}+r_{1}\theta_{1}-r_{2}\theta_{2}-e_{12}\left(\theta_{1},\theta_{2}\right)\\ e_{12}\left(\theta_{1},\theta_{2}\right)=E_{12}\sin\left(Z_{1}\theta_{1}+\zeta_{12}\right)+E_{1}\sin\left(\theta_{1}+\eta_{1}\right)+E_{2}\sin\left(\theta_{2}+\eta_{2}+\alpha_{12}\right) \end{array}\right.$$

The variable r_i_ denotes the base circle radius of gear i (where i = 1, 2); θ_i_ represents the rotational angle of the gear; k_12_, C_12_, e_12_, and α_12_, respectively, signify the time-varying mesh stiffness, mesh damping, cumulative mesh error, and mesh angle of the gear pair. k_xi_, k_yi_, c_xi_, and c_yi_ correspond to the radial support stiffness and damping in the x and y directions for gear i; T_i_ refers to the torque acting on gear i. E_12_ and E_i_, respectively, represent the amplitudes of the meshing frequency error for the gear pair and the rotational frequency error of gear i; ζ_12_ and η_i_ are the initial phases of the meshing frequency error and rotational frequency error, respectively; δ_12_ is the meshing deformation on the tooth surface engagement line considering the comprehensive error.

The equations of motion for the cutting motor model, the cutting drive system dynamic model, and the drum load model are compiled and organized into matrix form. This yields the electromechanical coupled system dynamics mathematical model for the cutting section, as shown in Equation (9).

(9) $$M\;\ddot{X}+\left(C_{m}{+C}_{t}\right)\;\dot{X}+\left(K_{m}{+K}_{t}{+K}_{b}\right){X=T}_{L}{+T}_{e}$$

In the equation, X represents the generalized coordinate vector, with X = [x_i_ y_i_ θ_i_], where i = m, 1, 2, …, 5, d; M, T_L_, and T_e_ are the generalized mass matrix, system load vector, and the electromagnetic torque vector of the cutting motor, respectively. K_m_, K_t_, and K_b_, respectively, represent the mesh stiffness matrix, torsional stiffness matrix, and bearing stiffness matrix, respectively; C_m_ and C_t_ represent the mesh damping matrix and torsional damping matrix, respectively. The equations in Equation (9) describe a system of second-order differential equations for the mechanical transmission system. For ease of computation, it is first necessary to reduce the order, transforming it as follows:

(10) $$\left\{ \begin{array}{ccc} \frac{d}{dt}X & = & \overset{˙}{X}\\ \frac{d}{dt}\overset{˙}{X} & = & -M^{-1}(C_{m}+C_{t})\overset{˙}{X}-M^{-1}(K_{m}+K_{t}+K_{b})X\\ & & +M^{-1}(T_{L}+T_{e}) \end{array}\right.$$

This can further be expressed in matrix form:

(11) $$\begin{array}{ccc} \left\{\begin{array}{l} \dot{X}\\ \ddot{X} \end{array}\right\} & = & \left[\begin{array}{cc} I & 0\\ -M^{-1}\left(C_{m}+C_{t}\right) & -M^{-1}\left(K_{m}+K_{t}+K_{b}\right) \end{array}\right]\left\{\begin{array}{l} X\\ \dot{X} \end{array}\right\}\\ & & +\left\{\begin{array}{c} 0\\ M^{-1}\left(T_{L}+T_{e}\right) \end{array}\right\} \end{array}$$

**Figure 21:**
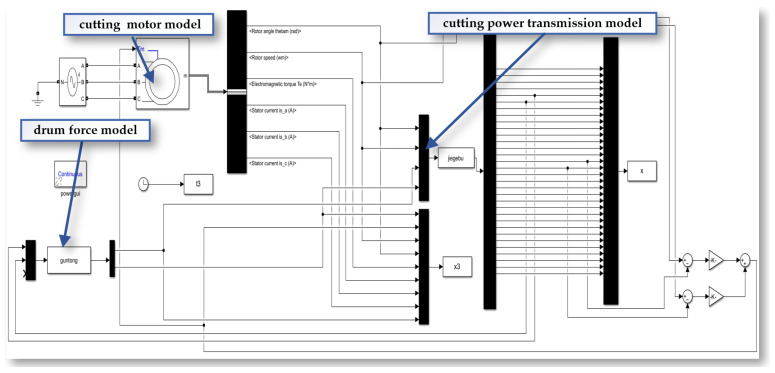
The mechanism model of the cutting experiment platform.

Based on the aforementioned mathematical model, simulation models of the cutting motor and the cutting drive system are constructed separately on the MATLAB/Simulink (R2022b) platform, as shown in Figure 21. The angular displacement and angular velocity of the cutting motor are used as shared variables to transfer data between the cutting motor and the cutting drive system. This data is then used to calculate in real time the torsional load on the motor output shaft, which is directly fed back to the cutting motor. Consequently, this process establishes an electromechanical coupled simulation model for the cutting drive system.

During computation, the ode45 solver provided in MATLAB is utilized (employing the fourth- and fifth-order Runge–Kutta method, which uses a fourth-order method to generate candidate solutions and a fifth-order method to control errors, constituting an adaptive step-size numerical solution technique for ordinary differential equations). This allows for the solving of the system’s differential equations, thereby obtaining the dynamic response of each component of the system.

### 4.3. Data Layer

The data layer involves the composition of data and its flow process. Its core functions include data management, data forwarding, data storage, and data collection. Data management, as the foundation of the data layer, includes the organization, cleaning, and transformation of data, and ensuring data quality and consistency. Data forwarding is mainly responsible for transferring data from one part of the system to another, such as from a storage system to an application server. Data storage focuses on storing collected data in databases for subsequent querying, analysis, and processing. Data collection is the process of acquiring data from various sensor terminals.

The composition of the data layer includes coal rock sample model data, cutting experimental platform model data, algorithm model data, and sensor data. Coal rock sample model data refers to the attribute data used to create three-dimensional models and predict and analyze the characteristics of coal rock samples. Cutting experimental platform model data involves the parameters of the three-dimensional model of the cutting experimental platform, which are used to build the simulation model of the cutting process. Algorithm model data is generated by processing and analyzing collected data through machine learning or other data analysis methods, producing data used for prediction or decision support. Sensor data refers to the information collected in real time from experimental platform sensors, which is crucial for monitoring and controlling the cutting process. In summary, the data layer spans the entire process from data collection and processing to application, forming a complete data management and analysis system.

**Figure 22:**
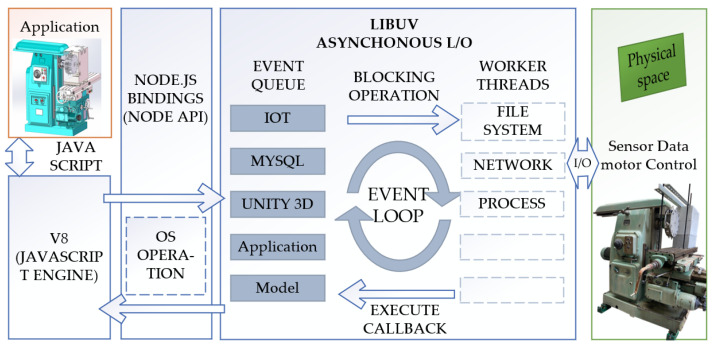
The architecture and process of a data application developed using Node.js.

Figure 22 illustrates the architecture and process of a data application developed using Node.js (v20.13.1) which, with its event-driven and non-blocking I/O capabilities, can efficiently handle a large number of concurrent operations, making it highly suitable for web applications and systems that require high-performance I/O operations. The entire architecture is based on an event-driven and non-blocking I/O model to optimize performance and concurrency handling.

Node.js is used to build WebSocket services for different applications. It is a server-side JavaScript runtime environment based on Google’s Chrome V8 engine, capable of executing JavaScript code. Node.js inherently supports TCP/IP and HTTP protocols, while building WebSocket protocols with Node.js requires the additional use of the core HTTP Server library provided by Node.js. This article has chosen the WS library for construction, which can be directly downloaded and installed using NPM. Node.js is not limited to server-side operations but can also be used in the Internet of Things (IoT). Applications can interact with the physical space of the real world, such as collecting sensor data and controlling motors through I/O operations.

The core of the software is the Event Loop, supported by the libuv library. libuv is a library specially designed for asynchronous I/O operations, which works across platforms and plays a key role in Node.js. The Event Loop is responsible for coordinating all asynchronous operations in the program. The Event Queue lists various types of operations waiting to be processed, including the Internet of Things (IoT), databases (MySQL), three-dimensional engines (Unity 3D), application logic layer (Application), and data model layer (Model). These operations are queued and wait for the Event Loop to process them in order. Worker threads are responsible for handling operations that may block the Event Loop. In Node.js, these operations are usually performed through built-in modules or extension modules, such as file system access, network requests, or executing some CPU-intensive processing tasks.

### 4.4. Digital Twin System Interaction Interface

**Figure 23:**
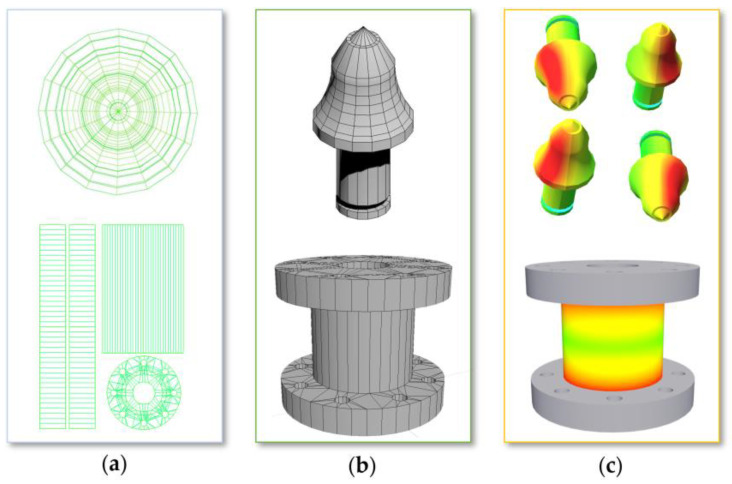
(**a**) Expanded UV grid; (**b**) UV mapping; (**c**) heat map rendering.

Experimental platform digital twin systems are created using Unity3D (2020.3.43 f1c1), where three-dimensional models are imported and then driven based on sensor data. The steps for importing 3D models are as follows: First, prepare the model file and use 3ds Max (20.2.0.2320) software to convert the experimental platform model into an fbx format supported by Unity3D, as shown in Figure 23.

Then, drag the model file into the Assets folder of the Unity3D editor to complete the import of the model file. Unity3D will automatically process the imported model. Next, drag the imported model from the Assets folder to the scene, and adjust the model’s position, rotation angle, and scale to meet the requirements of the scene.

**Figure 24:**
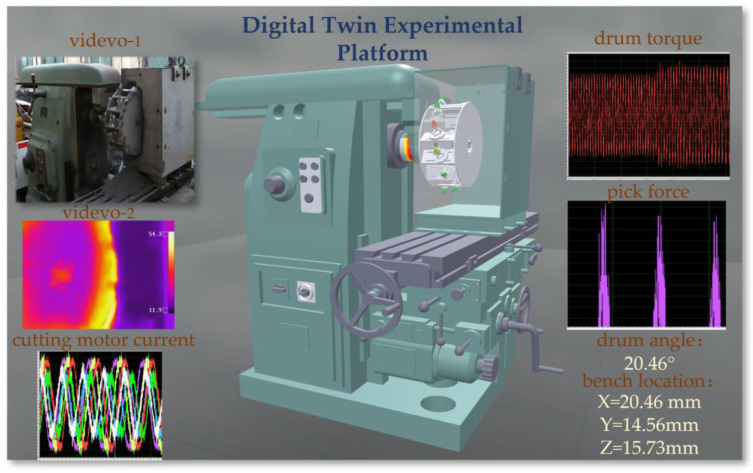
Digital Twin Experimental Platform System Interaction Interface.

Programming is required to process sensor data. First, determine the type and interface of the sensor used. In Unity3D, C# scripts can be written to read sensor data. In addition, the WebSocket library’s API is used to read sensor data transmitted over the network. Based on the obtained sensor data, Unity3D’s Transform component is used to adjust the position and rotation angle of the experimental platform model. The digital twin experimental platform’s system interaction is shown in Figure 24.

The experimental platform digital twin system also integrates visible light and infrared video camera streams. By using Unity’s WebCamTexture class, a WebCamTexture object can be created, and the camera’s name specified. Then, assign the WebCamTexture object to the Material’s Texture property to display the video on a GameObject in the scene. In addition, control of the WebCamTexture’s playback and pause is required.

The system uses the LineRenderer component to display a dynamic curve graph of real-time sensor data. The operation process is as follows: Create an empty GameObject in the Unity editor and add a LineRenderer component to it. Then, write a new C# script to control the updating and rendering of the curve data. In the script, calculate the position of each point on the curve based on the dynamically updated data, and use the LineRenderer’s SetPositions() method to update these points, thereby achieving dynamic updating of the curve.

## 5. Simulated Cutting Experiments

### 5.1. Preparation of Simulated Coal Sample

**Figure 25:**
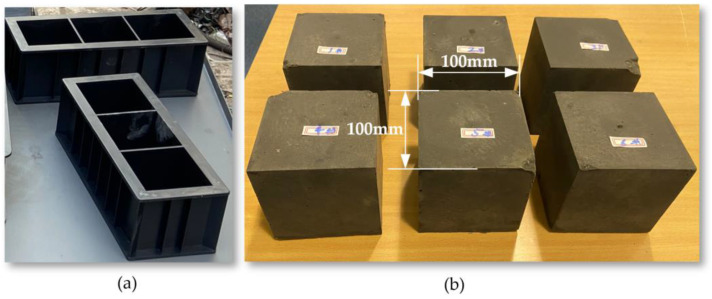
(**a**) Coal Rock Specimen Test Mold; (**b**) Coal Rock Experimental Specimen Block.

In this experiment, the mass ratios of coal dust, cement, sand, and water were used as control variables to study the variation in compressive strength. Initially, the simulated coal samples were prepared for each experimental scheme, as shown in Figure 25. The preparation process included the following steps: first, coal dust, sand, and cement were sifted through a 20-mesh screen. Then, according to the established experimental scheme, coal dust, cement, sand, and water were measured in sequence and thoroughly mixed and stirred evenly. The mixed materials were then filled into molds and compacted to form.

The standard experimental coal column calibration samples used 100 mm cubic columns. Two days later, after the samples had basically solidified, demolding and labeling were performed. After demolding, calipers were used for measurement. Because the initial compression area has a significant impact on the actual compressive strength results during the compression process, it is necessary to ensure the parallelism of both ends adequately. The coal column was placed horizontally on the platform, and a dial indicator was used to collect the height, ensuring the coal column’s surface was smooth to avoid stress concentration.

**Figure 26:**
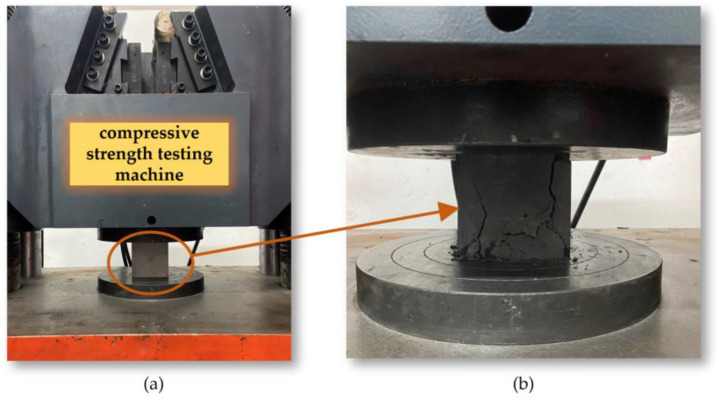
(**a**) Concrete Compressive Strength Testing Machine; (**b**) Tensile Strength Test.

**Figure 27:**
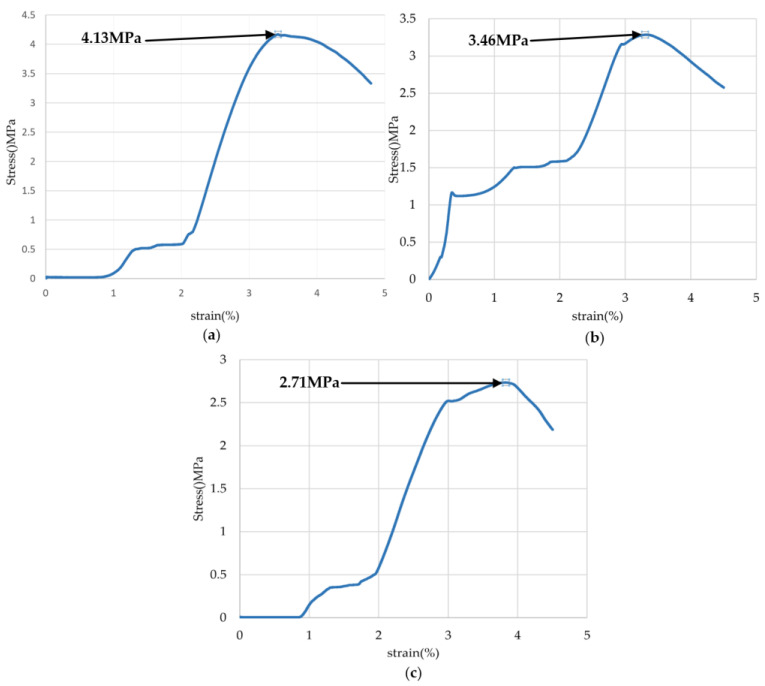
Stress–strain curves of simulated coal samples (**a**) 4.13 MPa; (**b**) 3.46 MPa; (**c**) 2.71 MPa.

The simulated coal samples needed to be cured at room temperature for 14 days. After curing, the calipers and electronic balance were used for measurement and weighing, the density of the materials was calculated and recorded. The experiment used a 20 kN microcomputer-controlled electronic universal concrete compressive strength testing machine to test the uniaxial compressive strength of the simulated coal samples, as shown in Figure 26. The experiment chose a displacement-controlled load application mode and set the loading rate at 1.5 mm/min. As the test machine gradually increased the given load, the simulated coal samples were pressed until destruction, and the compressive strength was recorded, as shown in Figure 27.

**Figure 28:**
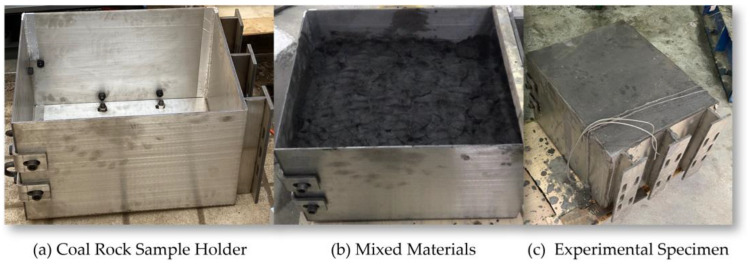
Injection of Mixed Materials into Coal Rock Sample Holder.

Finally, the experimental data were averaged to obtain the average compressive strength, and according to the mass ratios of coal dust, cement, sand, and water, the sample was poured into the coal rock holder designed for the experimental platform, as shown in Figure 28.

The experimental cutting test conditions of the test platform were set as follows: the simulated drum speed was set to 60 r/min, the translation speed of the cutting sample was 0.5 m/min, the drum outer edge diameter was 385 mm, the pick installation angle was 40°, and the pick inclination angle was 0°. This experiment aims to conduct cutting tests on three samples with different hardnesses, which are:

- Experimental mode one—cutting ratio simulated coal seam material, with a compressive strength of 2.71 MPa and a density of 1388.46 kg/m^3^;
- Experimental mode two—cutting ratio simulated coal seam material, with a compressive strength of 3.46 MPa and a density of 1506.56 kg/m^3^;
- Experimental mode three—cutting ratio simulated coal seam material, with a compressive strength of 4.13 MPa and a density of 1658.45 kg/m^3^.

### 5.2. Time Domain Analysis of Sensor Signals

#### 5.2.1. Analysis of Infrared Thermal Imaging

**Figure 29:**
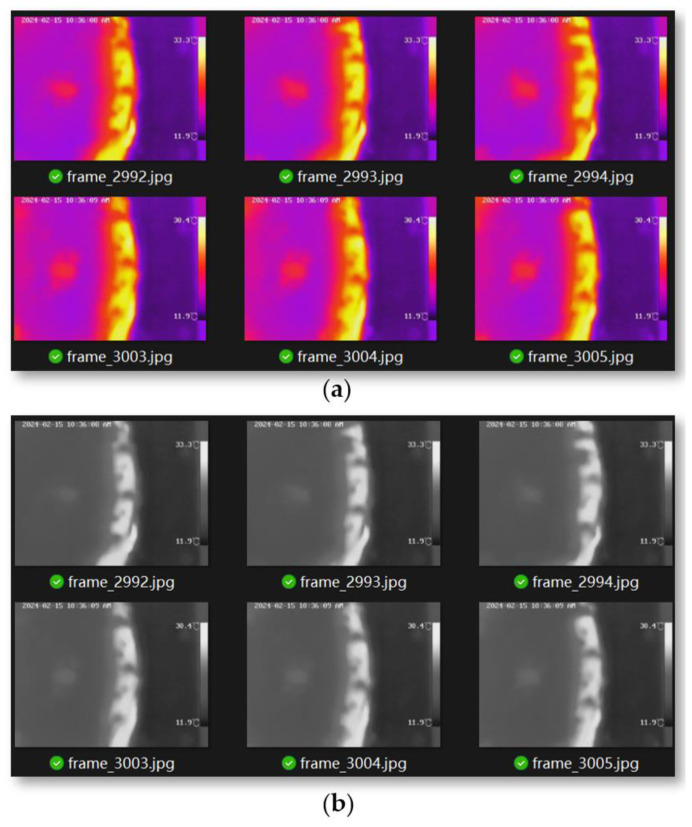
(**a**) Infrared thermal false color imaging; (**b**) infrared thermal grayscale imaging.

Infrared thermal imaging typically uses false colors to represent different temperature intervals, creating an intuitive visual representation that shows the relative temperature distribution in different areas of the image. However, this method cannot directly provide accurate temperature measurements and requires further analysis through algorithmic processing. In contrast, grayscale images intuitively display temperature information using black and white colors, with brightness variations from white to black indicating the spectrum from high to low temperatures. As shown in Figure 29, converting the color image of infrared thermal imaging into a grayscale image and then calculating the temperature is an effective method. This process includes two steps: first converting the color image into a grayscale image, and then deriving the temperature values based on the grayscale levels.

The grayscale value of a pixel is linearly related to a certain range of temperatures. Therefore, the first step involves converting a color image into a grayscale image. This conversion is achieved by calculating the weighted values of the three channels of the color image, as demonstrated by the following equation:

(12) $$Y=0.299M_{r}+0.587M_{g}+0.144M_{b}$$

In the equation, Y represents the converted grayscale value, Mi represents the matrices of the extracted different color channels, where r, g, and b represent the red, green, and blue color channels, respectively.

During direct contact between the pick and coal rock samples, heat is generated due to the impact, compression, and friction between them, leading to a rise in temperature of the pick and its cutting area. When the translation speed of the cutting sample and the drum rotation speed are constant, the properties of the coal rock become the key factors affecting the temperature changes in the pick and coal rock wall. This means the temperature variations in the pick and coal rock wall after cutting will also differ. At the beginning of the cutting phase, the temperature of the contact surface between the pick and coal rock sample gradually increases. As the cutting progresses, the thickness of the cut by the pick increases, leading to a rapid rise in temperature of the cutting surface. At this point, the rate of heat exchange between the cutting surface and air also accelerates. Since the pick during the cutting phase is embedded in the coal rock sample, the infrared thermal imaging system cannot capture the real-time temperature of the pick. However, when the pick rotates out of the coal rock sample with the drum, the infrared thermal imaging system can capture the highest temperature region on the pick.

**Figure 30:**
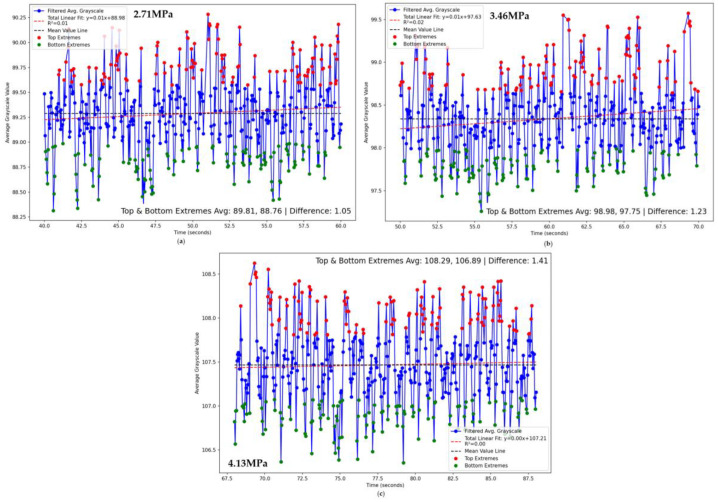
Grey value analysis of experimental images (**a**) Experimental mode one; (**b**) Experimental mode two; (**c**) Experimental mode three.

The purpose of this experiment is to measure the temperature changes in the cutting teeth when cutting samples of different hardness. In order to clearly capture the temperature information of the cutting teeth and reduce the interference of coal rock debris on temperature measurement, we reduced the advance speed of the coal rock samples. At the beginning of the experiment, the temperature changes rapidly; as the experiment progresses, the temperature changes gradually stabilize. At this point, the thermal imaging results are more representative. Figure 30 shows the grayscale change curve of the temperature after stabilization when cutting coal rock samples of different hardness. As the hardness increases, both the mean and fluctuation of the grayscale also increase. The figure averages the top 25% and bottom 25% of grayscale values, with fluctuations increasing by 17% and 14.6%, respectively.

#### 5.2.2. Analysis of the Force Sensor Signals

In the initial stage, as the cutting thickness of the coal sample by the cutter bit is relatively thin, the amplitude of the cutting force on the load–time domain curve is relatively small. With the continuous rotation of the drum, the cutter bit penetrates deeper into the coal sample, leading to a gradual increase in the instantaneous cutting thickness. This process is manifested as an increase in the amplitude of the cutting force on the load–time domain curve.

According to the pattern of cutting force fluctuation, a complete cutting cycle can be divided into four stages: the initial elastic deformation stage, the plastic deformation stage, the main crack formation stage, and the crack propagation stage. After the end of the crack propagation stage, the collapse of the coal block causes a sharp decrease in the cutting force. Once the cutting thickness reaches its maximum value, it will gradually decrease. During this process, the cutting load of a single cutter bit shows an overall trend of gradually decreasing from the maximum peak value until it finally exits the cutting and enters the no-load stage. The change in load during this stage is primarily due to the gradual decrease in cutting thickness caused by the rotation of the drum, which in turn reduces the corresponding cutting load.

**Figure 31:**
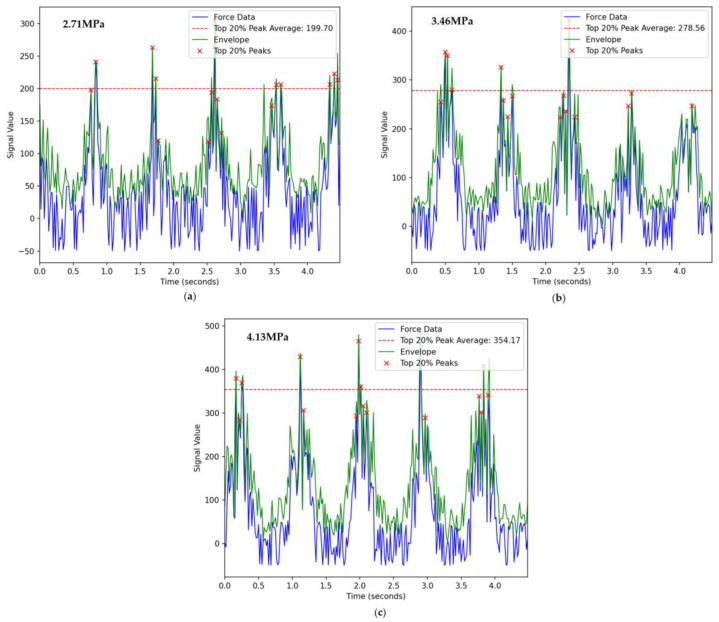
Experimental pick force analysis (**a**) Experimental mode one; (**b**) Experimental mode two; (**c**) Experimental mode three.

The calibration results depict sensitivities of 0.748 mV/V, 2.367 mV/V, and 2.83 mV/V for the three-dimensional force [17], respectively. Furthermore, the cross-sensitivity error was lower than 5.02%. The cutting load Fi can be solved by means of the coupling matrix K. Figure 31 averages the top 20% of force values, with fluctuations increasing by 40% and 27.6%, respectively.

#### 5.2.3. Analysis of the Torque Sensor Signals

**Figure 32:**
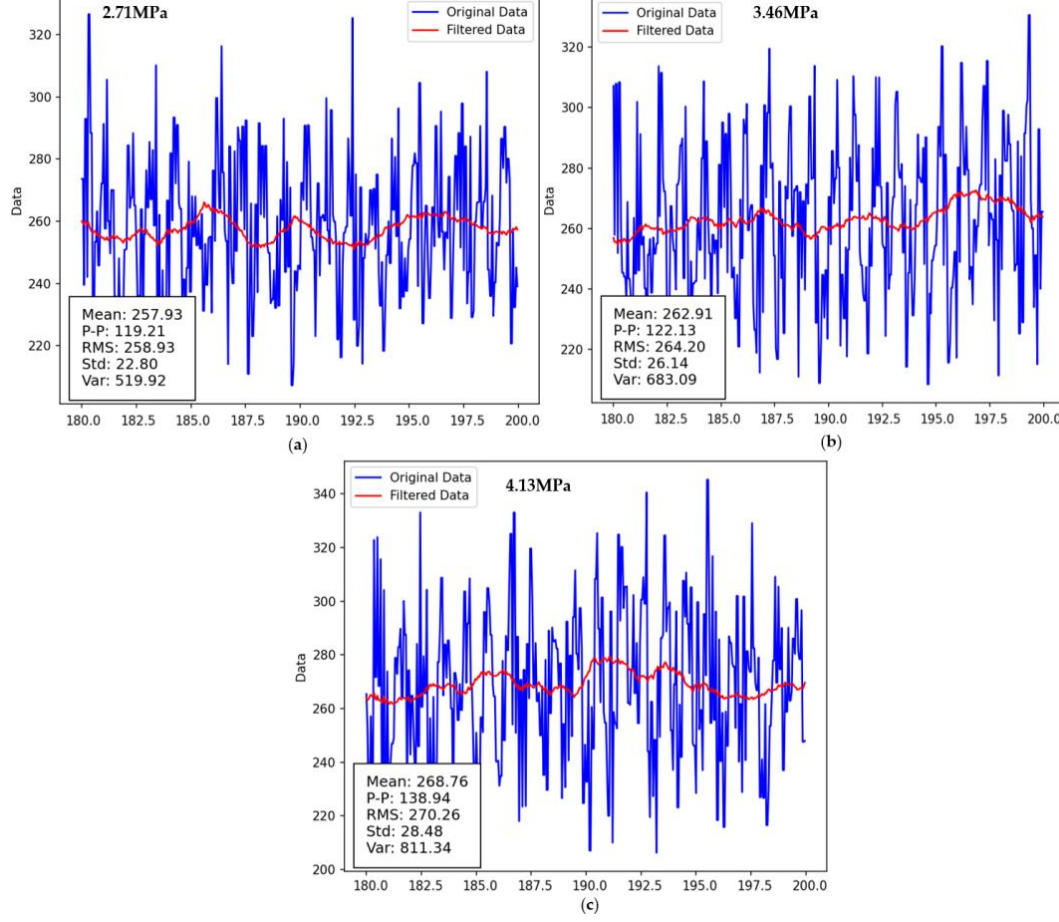
Experimental torque value analysis (**a**) Experimental mode one; (**b**) Experimental mode two; (**c**) Experimental mode three.

The torque fluctuations in drum cutting are relatively strong, and the load changes are irregular, with load peaks varying significantly. This is closely related to the number of cutting teeth involved in the cutting process and the properties of the material being cut. Different cutting teeth have different cutting entry angles and cutting thicknesses at the same moment, so it is not feasible to study the overall drum cutting load using a simple direct proportionality based on a single cutting tooth form. During drum cutting, it is not just an ideal scenario of cutting teeth engaging with the material; other components on the drum also come into contact with the specimen. The cumulative load from these components significantly affects the drum load. Figure 32 shows the torque sensor values from experiments with three different cutting modes. It is very difficult to distinguish between the three cutting modes based on these time domain indicators.

## 6. Conclusions

This paper elaborates on the design and experimental validation of a digital twin cutting experiment system for a shearer, focusing on simulating the cutting process for coal and rock identification. This encompasses the development of a simulated shearer drum based on the principle of similarity theory, the establishment of a comprehensive experimental platform, and the application of digital twin technology to bridge the gap between physical experiments and digital simulations. The key components of the study include:

The design of a simulated shearer drum—employing similarity theory to ensure the simulated drum accurately mirrors the cutting actions of a real coal mining machine, thus enhancing the reliability of the simulation experiments.

The experimental platform device structure—modifying existing machinery to meet experimental requirements, including the integration of sensors and a data acquisition system for real-time monitoring and analysis.

The software system design in digital space—developing a digital twin that comprises data layers for management and analysis, models for simulation, and application layers for interactive experimentation and algorithm training.

The simulated cutting experiments—performing tests with prepared coal samples to collect data on various physical forces, torque, thermal imaging, vibration, and sound, aimed at analyzing the cutting process and improving efficiency and safety in coal mining operations. By conducting time domain analysis of sensor signals collected during the cutting of materials of different strengths, it was found that the characteristics of the cutting force signal were the most distinct. Extracting the cutting force sensor signal as a characteristic value can effectively distinguish various cutting modes, providing a reliable experimental solution for coal rock identification research.

## Figures and Tables

**Table 1:** Parameters of the simulated shearer cutting system.

Parameters	Symbols	Units	M	L	T
Drum diameter	D	mm	0	1	0
Blade outer edge diameter	D_y_	mm	0	1	0
Drum cut depth	B	mm	0	1	0
Spiral blade lift angle	α_y_	°	0	0	0
lead of the blade	L	mm	0	1	0
Blade head number	Z	null	0	0	0
Blade pitch	S_y_	mm	0	1	0
Blade hub wrap angle	β_y_	°	0	0	0
Pick pitch	T_c_	mm	0	1	0
Pick installation angle	γ	°	0	0	0
Pick inclination angle	λ_s_	°	0	0	0
Rotating speed of drum	n	r/min	0	0	−1
Traction speed	v	m/min	0	1	−1
Density	ρ	kg/m^3^	1	−3	0
Compressive strength	σ	Mpa	1	−1	−2

**Table 2:** Dimensional matrix of spiral drum design parameters.

Parameters	n	ρ	D	v	σ
Index	a1	a2	a3	a4	a5
M	0	1	0	0	1
L	0	−3	1	1	−1
T	−1	0	0	−1	−2

**Table 3:** Similarity coefficients of the simulated shearer cutting system.

Prototype	Model	Prototype	Model
D	D/3	Tc	Tc/3
D_y_	Dy/3	γ	γ
B	B/3	λs	λs
α_y_	αy	n	$\sqrt{3}$n
L	L/3	v	v/$\sqrt{3}$
Z	Z	ρ	ρ
S_y_	Sy/3	σ	σ/3
β_y_	βy		

## Data Availability

The data presented in this study are available on request from the corresponding author.
